# Pre- to Postbiotics: The Beneficial Roles of Pediatric Dysbiosis Associated with Inflammatory Bowel Diseases

**DOI:** 10.3390/microorganisms12081582

**Published:** 2024-08-02

**Authors:** Roberta Ottria, Ornella Xynomilakis, Silvana Casati, Pierangela Ciuffreda

**Affiliations:** Dipartimento di Scienze Biomediche e Cliniche, Università degli Studi di Milano, 20157 Milan, Italy; ornella.xynomilakis@unimi.it (O.X.); silvana.casati@unimi.it (S.C.); pierangela.ciuffreda@unimi.it (P.C.)

**Keywords:** dysbiosis, gut microbiota, infant, prebiotics, probiotics, synbiotics, paraprobiotics, postbiotic

## Abstract

Probiotics are “live microorganisms which, when administered in adequate amount, confer health benefits on the host”. They can be found in certain foods like yogurt and kefir and in dietary supplements. The introduction of bacterial derivatives has not only contributed to disease control but has also exhibited promising outcomes, such as improved survival rates, immune enhancement, and growth promotion effects. It is interesting to note that the efficacy of probiotics goes beyond the viability of the bacteria, giving rise to concepts like paraprobiotics, non-viable forms of probiotics, and postbiotics. Paraprobiotics offer various health benefits in children with intestinal dysbiosis, contributing to improved digestive health, immune function, and overall well-being. In this review, the potential of these therapeutic applications as alternatives to pharmacological agents for treating pediatric intestinal dysbiosis will be thoroughly evaluated. This includes an analysis of their efficacy, safety, long-term benefits, and their ability to restore gut microbiota balance, improve digestive health, enhance immune function, and reduce inflammation. The aim is to determine if these non-pharmacological interventions can effectively and safely manage intestinal dysbiosis in children, reducing the need for conventional medications and their side effects.

## 1. Introduction

The human microbiota plays a critical role in maintaining health and serves as a valuable resource for host defense. The early years of life are critical for the proper development of the gut microbiota, which is shaped by various factors, including the mode of feeding and delivery. The human gut contains 10^14^ bacteria and many other microorganisms, such as Archaea, viruses, and fungi, which are ten times more numerous than the total number of human cells. More than 95% of the gut microbiota can be classified into four major groups: *Firmicutes*, *Bacteroidetes*, *Actinobacteria*, and *Proteobacteria* [1]. After bacterial colonization in infancy, the intestinal microbial composition continues to develop and reaches its adult composition by 2–3 years of age, becoming unique to everyone, with a vast difference between health and disease [2,3].

Intestinal dysbiosis, an imbalance or disruption in gut microbial populations, due to quantitative or qualitative changes in composition and/or metabolic activities and distribution [4], is correlated to a plethora of gastrointestinal (GI) pathological conditions [5], such as inflammatory bowel disease (IBD) [6], *Helicobacter pylori* infection [7,8], celiac disease [9], nutritional disorders [10], and many other gastrointestinal disorders [11]. Intestinal dysbiosis is also observed in allergic conditions like atopic dermatitis, allergic rhinitis, asthma [11], obesity [12], and autism [13,14]. So, the most worrying question is what would be the origin of gastrointestinal dysbiosis? Specifically, Bajinka et al. [15] reviewed extrinsic factors that influence gut microbiota with positive or negative effects and, in some cases, are associated with intestinal dysbiosis, including dietary (high fat, fiber, animal fat, amino acids, gluten, sucralose), diet therapy (Mediterranean, Western, or Vegan diet), antibiotics and drugs use, oxidative stress, and socioeconomic status [16,17]. Certainly, the use of biotics, the focus of this review, influences the composition and activity of the gut microbiota and, if prolonged or incorrect can contribute to intestinal dysbiosis. 

**Figure 1:**
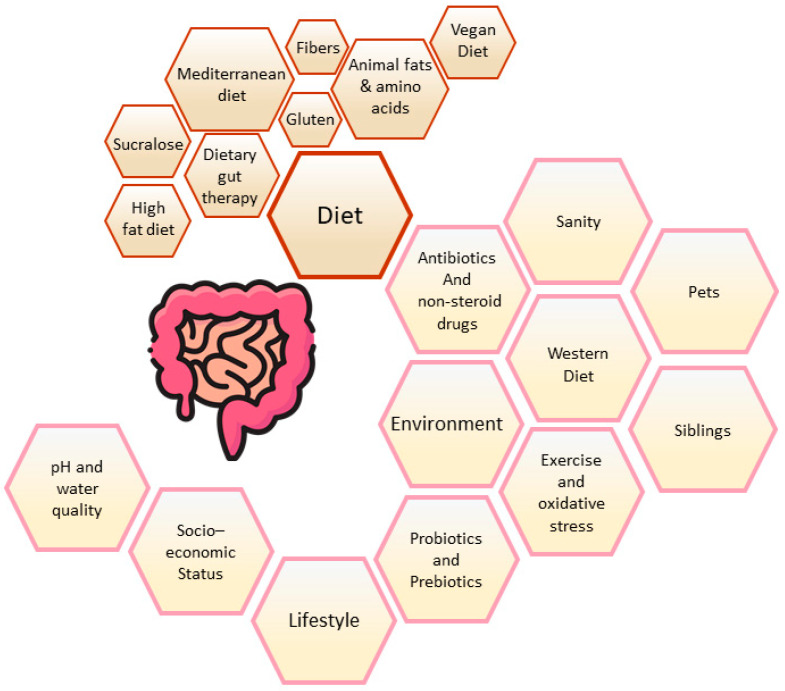
Factors causing alteration to gut microbiota.

The differences in the diversity and development of the gut microbiota in infants may also be explained by other factors. Among them, home biodiversity (family size and exposure to high levels of endotoxin in house dust) [18], the surrounding environment [19], the presence of siblings and family members [20], the environment while growing up (i.e., urban vs. rural), geographical location/population, and hygienic practices can impact microbial diversity [21]. In a recent study examining the Canadian population, it was reported that there has been a notable increase in IBD intestinal dysbiosis among children aged 0–5 years, while the incidence has remained steady in children aged 6 years and above [22]. Data from Asia and South America also suggest a similar trend, albeit with fewer patients overall [23]. The role of bacteria, fungi, and viruses in the development of IBD has been widely demonstrated and extensively reviewed elsewhere [14]. Moreover, since children are considered the best model to study the pathogenesis of IBD [15], our review aims to provide as much detail as possible in pediatric age. Figure 1 summarizes the potential factors contributing to gut dysbiotic conditions.

### Development of Infant Intestinal Microbiota

**Figure 2:**
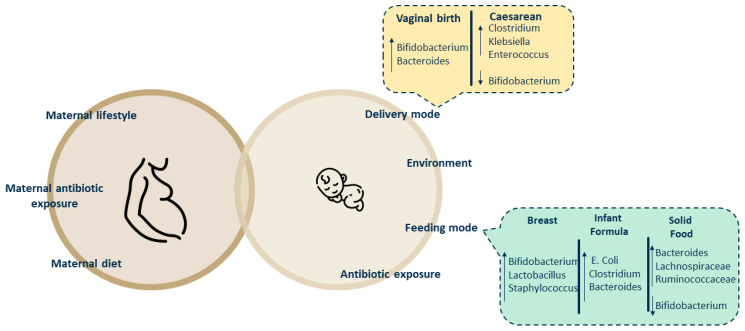
From in utero to post-natal life: factors that have been shown to affect the establishment of the gut microbiota.

Alterations in infants’ gut microbiota composition during their first year of life are influenced by various factors, and delivery mode and cessation of breastfeeding are two key factors [24,25] (Figure 2). Infants delivered by cesarean section (CS) indeed were found to have abnormal gut microbiota characterized by an increased presence of *Clostridium*, *Clebsiella*, and *Enterococcus* and a decreased presence of *Bifidubacterium* in respect to vaginally delivered ones [26,27]. One of the possible causes of these alterations in newborns is the need for antibiotic consumption by mothers undergoing cesarean section [28,29]. Moreover, mounting evidence suggests that an irregular gut microbiota composition due to delivery mode influences not only the subsequent regulation of immune response [30] but also may be linked to several clinical conditions [31]. Increased risk of obesity [32], celiac disease, type 1 diabetes [33], and asthma [34] have indeed been reported in children born via CS. This may result from the lack of initial exposure to maternal vaginal and intestinal flora at birth, potentially leaving children vulnerable to various diseases due to alterations in immune system development [35].

Extensive research has focused on dietetic influence on infants’ gut microbiota, specifically on breast milk versus formulas [36]. Human milk (HM) oligosaccharides (HMOs) constitute the third most abundant component of breast milk, after lactose and lipids. By supplying these oligosaccharides, breastfeeding promotes the growth of specific *Bifidobacterium* and *Lactobacillus* species that utilize HMOs and dominate the ecosystem as long as the baby is predominantly breastfed [37]. HMOs are composed of glucose, galactose, N-acetylglucosamine, fucose, and sialic acid. From these five units, over 200 distinct HMO structures have been identified [37]. HMOs function as prebiotics by stimulating the growth of beneficial bacteria while preventing the binding of harmful bacteria to intestinal epithelial cells. Moreover, species in the gut that ferment sugars produce metabolites that may protect against infectious and immune-related diseases. Formula feeding, lacking HMOs and with higher protein content, promotes instead a diverse gut microbiota development with more opportunistic pathogens, such as *E. coli*, *Clostridium*, and *Bacteroides*, and a more proteolytic metabolism. However, as the child grows and changes the type of nutrition by introducing new foods, the gut microbiota becomes more diverse and stable, resembling that of an adult by around 3 to 5 years of age. The period between exclusive milk feeding and the transition to eating family foods is usually referred to as the complementary feeding (CF) period, typically spanning from 6 to 24 months of age [38]. The World Health Organization (WHO) recommends starting CF around 6 months of age. However, in certain countries, it is not uncommon to introduce other foods as early as 2 or 3 months of age [39]. Early initiation of CF, before 3 months of age, has been associated with various health risks that include increased susceptibility to GI and respiratory infections, obesity, and allergies. However, these risks might be more attributable to the shorter duration of breastfeeding rather than the introduction of complementary foods at an early age [40]. CF, by introducing dietary fibers and new protein sources, triggers a transformation in the gut microbiota—with increased *Bacteroides*, *Lachnospiraceae*, and *Ruminococcaceae* and a decreased presence of *Bifidobacterium*—and in its metabolism, transitioning from a milk-adapted state to a more mature and diverse adult-like community, characterized by higher levels of short-chain fatty acid-producing bacterial taxa [41].

**Figure 3:**
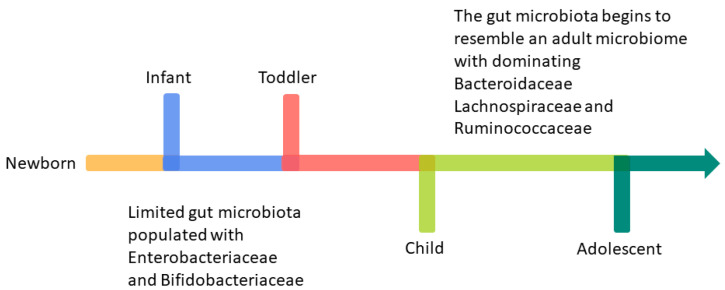
Change in gut microbiota with aging: dominant bacterial species found in human intestines during different stages of growth, from infancy to adolescence.

In early life, particularly infancy and early childhood, the gut microbiota undergoes significant changes and is more susceptible to disruptions compared to adults (Figure 3) [39]. Moreover, a reduced overall diversity in gut microbiota during infancy has been linked to allergic diseases [36,42]. Based on recent advances, the use of probiotics, prebiotics, or fermented dairy products to manipulate gut microbiota has been proposed to treat or prevent various disorders, including intestinal dysbiosis [43], also in early life. In this review, therapeutic applications of pre- to postbiotic-related products, which have the potential to be used as an alternative to pharmacological agents in the treatment of pediatric intestinal dysbiosis associated with IBD, will be investigated. 

## 2. Methods

**Figure 4:**
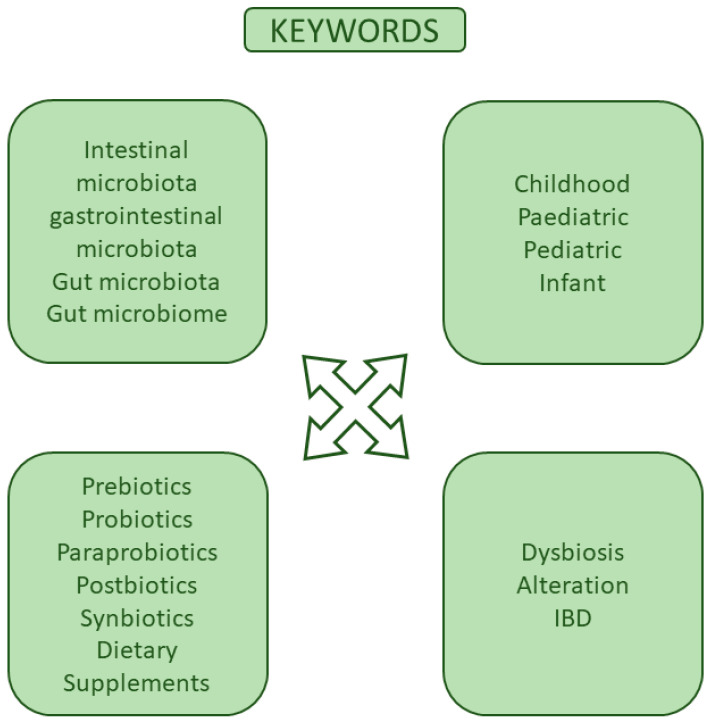
Principal keywords used for the literature search.

A rigorous electronic search was conducted on well-known scientific databases, including PubMed, Scopus, and Web of Science. The search was limited to the last 10 years, from 2014 to June 2024. The search was performed using a combination of keywords including pediatric, *biotics, and dysbiosis (Figure 4). Boolean operators and filters have been applied to refine and optimize the search results, improving the accuracy and relevance of the results. In a second step, more detailed keywords, for example, SCFAs or butyric acid, were added to the search. The articles were assessed according to the title, abstract, and full text. Additional references were included through meticulous examination of the initially reviewed articles, ensuring broad inclusion of both recent and prior literature. The extracted data were then reviewed and synthesized using a narrative approach, providing a detailed and coherent summary of the current state of the research.

## 3. Prebiotics, Probiotics, Synbiotics, and Paraprobiotics: Their Effects on IBD Intestinal Dysbiosis Affecting Children

### 3.1. Prebiotics

The concept of prebiotics was first introduced by Glen Gibson and Marcel Roberfroid in 1995. An initial description defines them as non-digestible food ingredients with beneficial effects for the host by stimulating the activity and/or growth of one or a limited number of host bacteria in the colon [44]. Since then, the concept has been refined, but its core principles have remained the same. According to the most recent review of International Scientific Association for Probiotics and Prebiotics, the current definition for “dietary prebiotics” is “a selectively fermented ingredient that results in specific changes in the composition and/or activity of the gastrointestinal microbiota, thus conferring benefit(s) upon host health” [45]. Furthermore, for an ingredient to be classified as prebiotic, it must exert its effect on a limited group of microorganisms and must comply with three aspects: be a substrate, have a physiologically beneficial effect, and have a microbiota-mediated mechanism. The effect exerted by prebiotics depends on their deployment by the host’s gut microbiota. This distinction separates prebiotics from other substances such as antibiotics, vitamins, bacteriophages, and minerals whose consumption alters gut microbiota composition. 

Thus, prebiotics must have the following characteristics:

- Indigestible by the host’s enzyme.
- Fermented selectively by gastrointestinal microbiota.
- Selectively promote the growth and/or activity of intestinal microbiota associated with health and well-being [45,46].

A large portion of prebiotics belongs to dietary fibers; nevertheless, not all have prebiotic properties. Some other main groups are inulin and fructo-oligosaccharide (FOS), galacto-oligosaccharides (GOS) including the family of raffinose oligosaccharides (RFO), trans-galacto-oligosaccharides (TOS), lactulose, resistant starch (RS), glucose-derived oligosaccharides such as polydextrose, and pectin oligosaccharides (POS). Prebiotics are naturally present in many plant-based foods, such as garlic, asparagus, beans, bananas, chicory roots, etc. [47,48], or synthetically produced to create food supplements [49]. Prebiotics reach the colon without being digested in the upper gastrointestinal tract [50] and exert their function by influencing gut microbiota through various mechanisms:

1. Fermentation: prebiotics are non-digestible but fermentable substrates that modulate bacterial taxa, enriching groups that utilize them [51].
2. Production of bioactive metabolites: the fermentation process yields bioactive metabolites such as SCFAs (butyrate, propionic acetic, and lactic acid) [52,53].
3. Modulation of bacterial composition: prebiotics stimulate beneficial bacterial species while potentially inhibiting harmful ones [54,55].
4. Cross-feeding: the metabolic action of prebiotics by some bacteria indirectly induces the growth of others [53,56,57].
5. Modulating gut environment: changes in pH levels of gut lumen as a consequence of fermentation products alter species composition [58].

Historically, *Lactobacilli* and *Bifidobacteria*, commonly known as probiotics, use prebiotics as substrates [44]. Data from high-throughput sequencing techniques reveal that prebiotics impact a broader range of microorganisms, but not all, in gut microbiota [56]. The gut microbiota acts as a homeostatic organ and participates in the fermentation of prebiotic substances, leading to the formation of bioactive metabolites and energy production with consequent beneficial effects on intestinal mucosa [59], protecting against pathogen colonization [60]. It is well-established in the literature that patients with intestinal dysbiosis associated with IBD exhibit increased colonization by harmful bacteria, which alters intestinal barrier permeability, giving rise to “leaky gut” syndrome and intestinal dysbiosis [61,62]. In this context, prebiotics contribute via the fermentation pathway, indirectly, to the enrichment of gut microflora with beneficent bacterial strains. These processes contribute to the formation of a microbiota that can overpower and/or resist intestinal dysbiosis and cause disease [63,64,65]. Moreover, the production of SCFAs as fermentation products of prebiotics also helps to maintain gut integrity [59,66]. 

Clinical studies on pediatric patients with IBD and the use of prebiotics are limited. HM is the first and an important source of prebiotics during pediatric nutrition. Studies demonstrate how the oligosaccharides contained in HM and HMOs, as described before, acting as prebiotic fermentable substrates, contribute to a healthy and balanced infant gut microbiota [67,68]. Newborn and infant supplementation with oligosaccharides to date is becoming usual due to the infant formula fortification and enrichment with these prebiotics, principally GOS, POS, and FOS [69,70,71]. However, formula fortification and enrichment with non-digestible carbohydrates is a research hot topic due to their beneficial effects on microbiota and gastrointestinal tract homeostasis and their functions in general [72,73]. Finally, SCFAs produced through fermentation of undigested oligosaccharides are associated with the maintenance of intestinal integrity and aid in blocking pathogens and toxins by binding to epithelial cells [74]. The correct development of the intestinal barrier during pediatric age is particularly important in premature infants, where there is a strong drive to develop intestinal dysbiosis and increased intestinal permeability, thereby increasing the risk of necrotizing enterocolitis [9]. 

### 3.2. Probiotics

**Figure 5:**
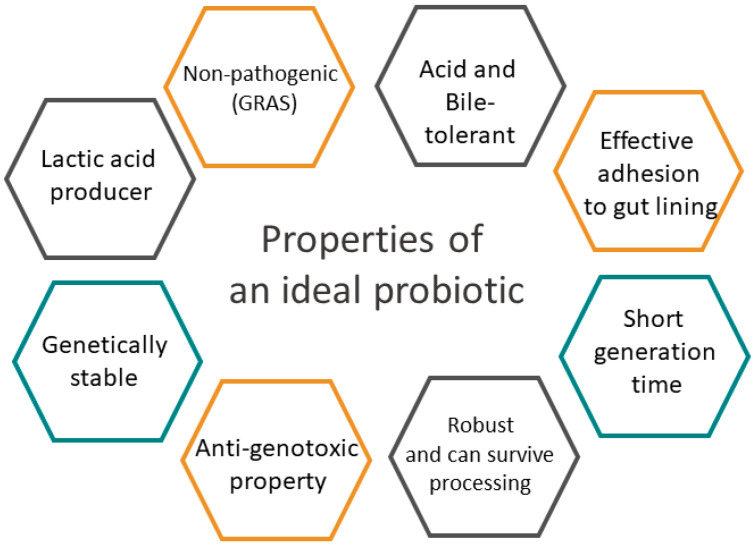
Characteristics of an ideal probiotic strain [76]. GRAS = Generally Recognized as Safe.

The term probiotics is derived from a Greek word meaning “for life” and is used to define living non-pathogenic organisms and their derived beneficial effects on hosts. The current definition, established by the Food and Agriculture Organization (FAO) and the WHO in 2001 and slightly modified by the International Scientific Association for Probiotics and Prebiotics (ISAPP) in 2014 describes probiotics as “live microorganisms which, when administered in adequate amounts, confer a health benefit on the host” [75]. This emphasizes the viability of microorganisms as crucial for the classification of probiotics (Figure 5). 

It is important to clarify the precise scope and optimal use of the term “probiotic” to ensure that scientists, industries, regulators, and consumers in the field of probiotics have a common understanding of what is currently known about probiotics, in line with the latest research. This clarification will facilitate the continued advancement of probiotic research and will guarantee that the benefits of probiotics are accurately conveyed to consumers and patients [75]. It is worth pointing out that the presence of live microorganisms in various foods and nutritional supplements is not uncommon. However, only those strains that have been subjected to rigorous scientific investigation and that have been found to exert a beneficial impact on human health can be considered probiotics. Microbes that are alive and present in traditional fermented foods and beverages typically do not meet the required evidence level for probiotics since their health effects have not been confirmed and the mixtures of microorganisms are largely uncharacterized. Fermented foods may be claimed as probiotics if they contain an adequately characterized live microorganism or strain at doses that may provide benefits documented by at least one positive human clinical trial conducted according to generally accepted scientific standards or, if applicable, local or national government recommendations and requirements. Finally, the microorganism must be safe [77]. The most prevalent genera of bacteria currently available as probiotics and exhibiting beneficial health effects include *Bifidobacterium*, *Lactobacillus*, *Bacillus*, *Enterococcus*, *Pediococcus*, and the yeast *Saccharomyces* [75,78,79]. 

Each genus contains numerous species, and each species comprises numerous strains. It is generally considered that the health effects of probiotics are strain-specific [80]. Probiotics are commercially available as single strains or in formulations in which more strains are present in different combinations [81]. It is therefore not feasible to define the mechanism by which probiotics exert their effects. Nevertheless, several common mechanisms have been identified. Probiotics can influence the composition of the gut microbiota, competing with pathogens for nutrients and binding sites on the intestinal wall, enhancing intestinal barrier function, and modulating the immune system [82]. Their capacity to produce antimicrobial substances and other metabolites such as SCFAs enables them to exert a direct or indirect effect on host health by lowering the pH [83]. In addition, these microorganisms have the capacity to affect the nervous system of the host through the gut–brain axis [84,85]. As evidenced by their ability to regulate the composition of the intestinal microbiota, probiotics have been implicated as a potential treatment option for a range of gastrointestinal conditions, including IBD, that lead to variations in the microbiota causing intestinal dysbiosis [46,86,87,88]. Probiotics have been significantly investigated regarding the management of inflammatory conditions. However, the evidence base in relation to the use of probiotics in the pediatric population with IBD is limited [89,90].

In accordance with the current evidence-based guidelines established by the European Crohn’s and Colitis Organisation (ECCO) and the European Society for Pediatric Gastroenterology, Hepatology, and Nutrition (ESPGHAN), VSL#3 and *Escherichia coli Nissle 1917* may be regarded as an efficacious treatment for maintenance in patients with ulcerative colitis [91]. However, this recommendation lacks sufficient evidence to be considered valid [92,93]. With respect to Crohn’s disease, the ECCO/ESPGHAN guidelines conclude that there is currently insufficient evidence to demonstrate that probiotics are beneficial for the induction or maintenance of remission. In summary, the available evidence indicates that selected probiotics may be useful in the treatment of ulcerative colitis but not in Crohn’s disease [94]. The clinical studies that support these conclusions and guidelines in the use of probiotics in pediatric IBD have recently been summarized [95,96]. To achieve the desired probiotic health effects, it is essential to administer probiotics in an adequate amount. The optimal dose of probiotics has not yet been clearly established and may vary depending on the specific probiotic strain, the condition being treated, and other factors. Until further data are available, it is advisable to utilize the established treatment regimen (probiotic dosage and formulation, duration of treatment), which has been demonstrated to be effective in well-designed and conducted randomized controlled trials (RCTs) for the specific indication under consideration [97].

Although there is evidence to support the use of specific probiotics in certain clinical contexts, further studies are frequently needed to confirm the effect of these probiotics, as well as to define the most suitable type, dose, and timing. As a general criterion, the administration of probiotics to children with no documented health benefits should not be recommended [98].

### 3.3. Synbiotics

Synbiotics are dietary supplements that combine probiotics and prebiotics in the form of synergism [99,100], increasing the beneficial effects of probiotics [101]. So, it should be reserved only for products in which the prebiotic compound selectively favors the probiotic organism [102]. According to the current understanding, there are two types of synbiotic approaches [103]:

- Complementary, where the selection of the probiotic is based on beneficial impacts intended for the host, while the prebiotic is selected separately to enhance the levels of beneficial microbial components. The prebiotic can support the growth and activity of the probiotic but does so indirectly as part of its broader target spectrum.
- Synergistic, in which the probiotic is again chosen based on specific beneficial effects on the host, but the prebiotic is chosen to stimulate specifically the growth and activity of the selected probiotic. In this case, the prebiotic is selected to have a higher affinity for the probiotic and is chosen to enhance its survival and growth in the host. It can also increase levels of microbiota beneficial in the host, but the main target is the ingested probiotic.

Studies have been conducted on the effects of synbiotics on the GI microbiota in infants using supplementation in infant formula [104,105]. At present, the available evidence is insufficient to support the regular inclusion of these “-biotics” in infant formulas for healthy or atopic infants who are unable to be breastfed and there is currently no strong evidence to suggest any benefits either. However, the use of synbiotics to treat intestinal dysbiosis in children remains theoretical. To the best of our knowledge, there is no documented use of synbiotics to treat intestinal dysbiosis in children.

### 3.4. Paraprobiotics

**Figure 6:**
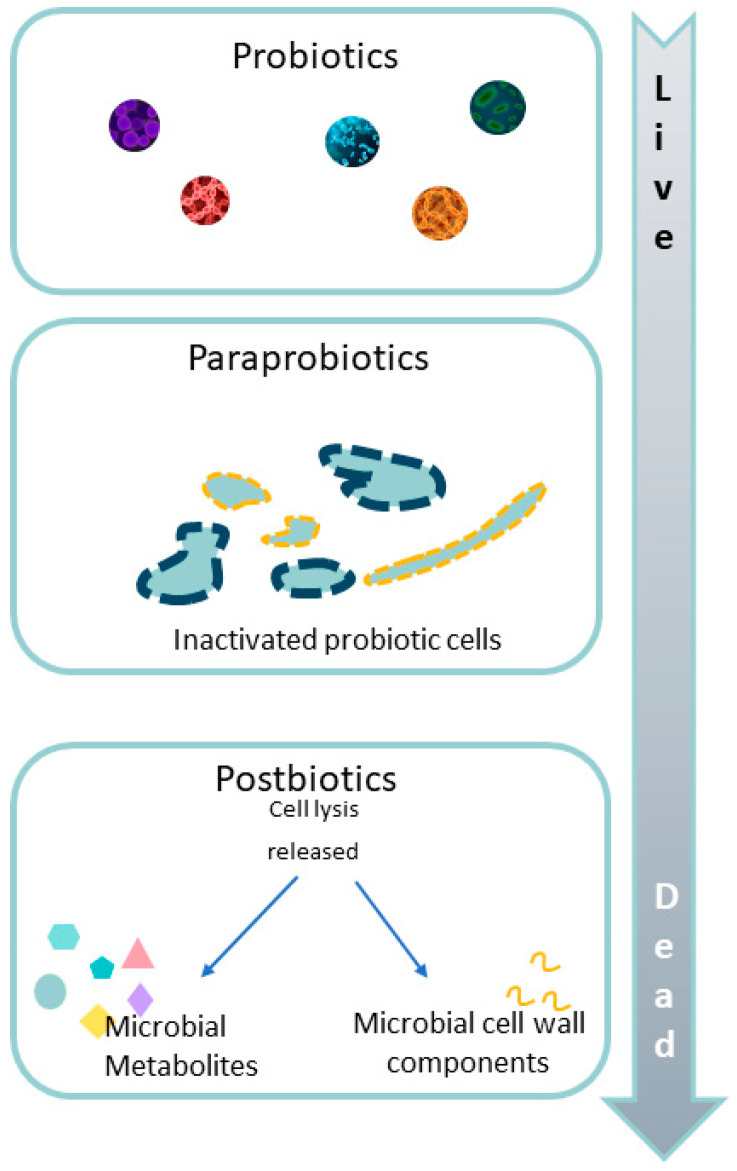
Conceptualization of paraprobiotics and postbiotics [109].

Paraprobiotics have been described using various terms, such as inactivated probiotics, ghost probiotics, and nonviable (dead) probiotics (Figure 6) [106]. Unlike traditional probiotics, which are live microorganisms, paraprobiotics include dead bacterial cells, cellular components, and cell lysate or microbial extracts with health benefits that are stable, safe for use, and confer advantages to the host [107]. As per the FAO/WHO, paraprobiotics are defined as inactivated (non-viable) microbial cells, which, when managed in sufficient amounts, present benefits to consumers [107]. Paraprobiotics can be used in various forms, including dietary supplements, functional foods, and pharmaceuticals. Research on paraprobiotics is still in its early stages, but studies have shown promising results in various health conditions, such as gastrointestinal disorders and inflammatory diseases [108]. To the best of our knowledge, like synbiotics, there is no documented use of paraprobiotics in the treatment of pediatric intestinal dysbiosis.

### 3.5. Next-Generation Probiotics

Next-Generation Probiotics (NGPs) refer to advanced formulations of probiotics that incorporate innovative approaches to enhance their effectiveness and specificity [110]. These formulations often include strains selected for their ability to survive stomach acid and bile salts, adhere to intestinal surfaces, and modulate gut microbiota composition. Additionally, they may include encapsulation technologies or prebiotics to improve delivery and viability. In this context, several commensal microbial species identified in the intestinal microbiota have been proposed as probiotics of NGPs. Among the most promising candidates are the strains of *Akkermansia muciniphila* species, *Faecalibacterium prausnitzii* (e.g., *Fusobacterium prausnitzii*), *Anaerobutyricum hallii* (e.g., *Eubacterium hallii*), as well as *Bacteroides* spp., *Roseburia* spp., *Clostridium butyricum* [111], *B. pseudopodium*, *L. rhamnosus*, *L. acidophilus*, and *L. lactobacillus*, which modulate the serotonergic system in IBD [112]. These commensal bacteria are associated with a healthy state of the intestinal microbiota when present in adequate quantities. The aim is to optimize health benefits by targeting specific health conditions or populations, such as those with gastrointestinal disorders or immune deficiencies. Research in this field continues to explore novel probiotic strains and delivery mechanisms to further improve efficacy and therapeutic outcomes. It can be stated that there is significant potential for NGPs to treat gastrointestinal disorders, such as IBD, that exert their therapeutic effects by outcompeting pathogenic bacteria, regulating gut motility, producing SCFAs, and improving gut permeability. However, further research is necessary to confirm the efficacy of NGPs in managing these conditions. As of today, the use of NGP in pediatrics is limited, and there is no application for the treatment of intestinal dysbiosis associated with IBD in the pediatric field.

## 4. Postbiotics, Metabiotics, Biogenics, or Simply Microbiota Metabolites: Beyond Probiotics and Prebiotics on Pediatric Intestinal Dysbiosis in IBD

Postbiotics, metabiotics, biogenics, inactivated microbial intact (nonviable) cells, inactivated probiotics, phantom probiotics, and microbiota metabolites are all the different names used for the “preparation of inanimate microorganisms and/or their components that confers a health benefit on the host” [108,113]. Postbiotics include SCFAs, tryptophan (Trp) metabolites, organic acids, bile acids, proteins, secreted enzymes, amino acids, neurotransmitters, vitamins, bacteriocins, terpenoids, biosurfactants, etc. [114,115]. Postbiotics have been defined as beneficial bioactive compounds originating from living organisms, commensal microbiota, in the gut environment during the nutrient fermentation process. With respect to living organisms (probiotics, synbiotics, etc.) [116] postbiotics possess some advantages in terms of stability, packaging, transportation, and storage. Knowing the specific structure of different postbiotics, they can be used in purified forms allowing for the elucidation of specific mechanisms of action and triggering only specific pathways. Moreover, the literature presents an application of postbiotic functional food production or the preparation of various commodities such as dairy foods, fish products, bread, vegetables, and meat due to their very low or potential non-toxicity [117,118]. A further advantage that makes them therapeutically attractive is the possibility to reach physiologically high concentrations together with their suitability for different administration ways following the principles of pharmacokinetics [119]. Their beneficial effects have been demonstrated not only on gut homeostasis and physiology, such as permeability, inflammation, oxidative damages, and immune response, but also on other tissues such as skin, oral mucosa, and the central nervous system (CNS) [120,121]. Finally, the safety profile of postbiotics over probiotics makes them suitable supplements also for newborns or infants, comprising the most vulnerable premature newborns or preterm newborns with compromised gut integrity and a high risk of probiotics sepsis [122,123,124]. Also in this regard, the postbiotic beneficial effects on infectious disease, inflammatory response [104,125], and gut morbidities such as colitis, enterocolitis, and necrotizing enterocolitis [118,126,127] have been demonstrated in newborns, infants, and children. From a biochemical point of view, the mechanistic aspects linked to the postbiotics biological effects are far from being elucidated. However, it is clear that numerous microbiota metabolites influence gut and brain functions such as metabolic and immune response or pain perception. A plethora of preclinical and clinical studies, indeed, support the concept of “ microbiota-gut-brain-axis ” and its physiological involvement in health and disease [128]. The gut–brain axis or more recently defined microbiota–gut–brain axis, is a complex communication system that links bidirectionally the gut and the nervous system comprising the central (CNS), autonomic (ANS), and enteric nervous systems (ENS) together with hormonal systems [129]. Increasing evidence supports the modulatory role of microbiota metabolites on different gut–brain axis pathways in IBD symptoms [130].

### 4.1. Short-Chain Fatty Acids (SCFAs)

**Figure 7:**
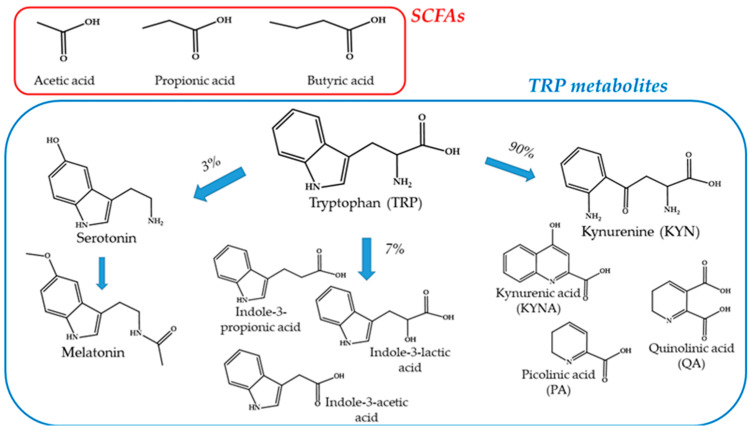
Short-chain fatty acids and tryptophan metabolites.

SCFAs, the products of saccharolytic fermentation of complex carbohydrates by intestinal microorganisms, especially acetate (60%), propionate (25%), and butyrate (15%) (Figure 7), were confirmed to reduce intestinal pH value and inhibit pathogenic microorganisms playing distinct roles in maintaining gut health and overall homeostasis [131]. Some different mechanisms of action have been proposed for SCFA contribution to gut health comprising nutrient supply for epithelial cells (butyrate) [132], gut motility regulation [133], and gut barrier restoration [134]. Different preclinical studies performed on cell models allowed for the proposal of SCFAs as key regulators of the inflammation process, affecting the recruitment and transmigration of immune cells [135]. Moreover, some mechanistic hypotheses have been formulated on their modulation of endothelial and immune cell inflammation through the binding to different intra and extra-cellular receptors [136,137] and the consequent control of pro- (IL-10) or anti-inflammatory (IL1β, IL12, TNF-α, IL-8) cytokine production [138,139,140,141]. In certain cases, direct supplementation of SCFAs may be used to restore their levels in the gut, helping to manage intestinal dysbiosis and its associated conditions, emerging as potential IBD treatment [142]. SCFAs play a vital role in mitigating the adverse effects of intestinal dysbiosis by maintaining gut barrier integrity, reducing inflammation, modulating the immune system, regulating metabolic processes, and influencing the gut–brain axis. Their therapeutic potential is being explored through various dietary and supplementation strategies to restore gut health and overall homeostasis in IBD in adults [143,144] and pediatrics [145,146]. Partial or exclusive enteral nutrition, performed with formulas containing low saturated fat, heme, and taurine and high protein, were demonstrated to be effective in inducing remission in children with Crohn’s disease, but reduced SCFA levels were found [147,148,149]. Moreover, an ex vivo experiment performed on circulating mononuclear cells from IBD patients and healthy controls showed a reduced sensitivity of the circulating immune cells from IBD patients to the anti-inflammatory effects of butyrate [150]. All these data support the need to investigate mechanistic aspects that link diet, microbial changes, and microbiota metabolite changes, leading to a reduction of gut inflammation in IBD.

Various clinical studies report a reduction in SCFA stool concentration in children with different nutritional states [151,152,153], pathological conditions [154,155,156], or gastrointestinal disorders [157,158,159]. However, only two recent clinical studies have reported on the use of SCFAs as supplements or add-on therapies in IBD for adults [160] and children [161]. Conducted across three pediatric centers specializing in the diagnosis and treatment of pediatric intestinal dysbiosis associated with IBD, this pioneering study involved 72 pediatric patients with a median age of 13.5 years. Participants received either 150 mg of sodium butyrate or a placebo orally in capsule form every 12 h for a duration of 12 weeks. The study found that sodium butyrate supplementation, at a total daily dose of 300 mg, was not effective as an adjunctive treatment for children and adolescents with IBD [137].

### 4.2. Tryptophan Metabolites

Tryptophan (TRP), a precursor of important bioactive amine, is an essential amino acid that is necessarily obtained from the diet. In the gut, TRP is mainly transformed in kynurenine (KNY, 90%) and serotonin (3%), with the remaining 7% being metabolized by the microbiota in indole derivatives (Figure 7) [162,163,164]. A dysregulation of TRP metabolism has also been associated with different pathological conditions such as obesity, metabolic disorders [165,166], and IBD, especially in Crohn’s disease patients [167,168], leading to an increased KYN/TRP ratio. This dysregulation is also supported by cytokines and cortisol levels and gut microbiota variations in microorganism composition and number during gut and neuroinflammation [169,170]. Between KYN second metabolites we can mention kynurenic acid (KYNA), quinolinic acid (QA), and picolinic acid (PA) with different effects on immune and inflammatory response [171,172]. While QA shows neurotoxic and pro-inflammatory effects [173], PA and KYNA [174] instead demonstrated neuroprotectivity, both in the central and enteric nervous systems, and anti-inflammatory properties [175], modulating immune cell differentiation and function [176]. KYN/TRP ratio dysregulation, moreover, could be linked to IBD mood comorbidities such as anxiety and depression by the consequent dysregulation of the serotonin pathway (Figure 6) [162]. Recent literature, considering preclinical and clinical studies, supports the promotion of KYNA as a potential pharmacological tool for IBD [177,178]. Together, these considerations suggest an in-depth exploration of the possibility of promoting KYN derivatives as a target signaling pathway for IBD hyperalgesia, such as for inflammatory bowel syndrome [179]. Microbiota metabolism of the last 7% of TRP introduced with diet leads to the production of protective indole derivatives, such as indole3-propionic, -lactic, -acetic acids, indole-3 acetaldehyde, and indole acrylic acids (Figure 6) [164]. Indoles are signaling molecules produced by the microbiota to regulate bacteria physiology. Additionally, these TRP metabolites may also support gut immune and anti-inflammatory responses and epithelial barrier functions, mediated by the activation of the aryl hydrocarbon receptor (AhR) [180]. The downregulation of AhR and reduced production of its ligands has been observed in IBD and intestinal dysbiosis [181]. The therapeutic potential of TRP metabolites is still emerging in the literature, and for this reason, clinical trials are still lacking even more so for pediatric patients. However, recent studies investigated KYN, KYNA, and TRP metabolites in neuroinflammation [182], autism [183], Kawasaki disease [184], and early childhood adiposity [185].

## 5. Other Supplements

### 5.1. Polyunsaturated Fatty Acids

There is evidence that suggests a relationship between ω-3 polyunsaturated fatty acids (PUFAs) and the gut microbiota. ω-3 PUFAs may alter the diversity and abundance of the gut microbiome just as the gut microbiota may influence the metabolism and absorption of ω-3 PUFAs. In addition, imbalanced consumption of n-3/n-6 PUFAs can lead to gut dysbiosis. ω-3 PUFA deficiency disrupts the microbiota community in metabolic disorders. ω-3 PUFAs include docosahexaenoic acid (DHA, C22:6), eicosapentaenoic acid (EPA, C20:5), α-linolenic acid (ALA, C18:3), and docosapentaenoic acid (DPA, C22:5). ω-3 PUFAs, the main source of which is fish oil [186], have a significant influence on immune homeostasis and modulation of the gut microbiota [187]. Various studies [187,188,189,190] have found that supplemental PUFAs, such as DHA and EPA found in fish oil, improve diet-induced microbiome changes (along with improvements in lipid profiles and fatty liver disease), with supplementation having a bigger effect on restoring the intestinal microbiome homeostasis and increased short-chain fatty acid production, which is both beneficial to health and also indicative of positive changes to the microbiome. Kaliannan et al. [191] indicated the beneficial effects of ω-3 fatty acids on antibiotic-induced gut dysbiosis and obesity, suggesting ω-3 supplementation as a safe and effective method for preventing obesity in children exposed to antibiotics. In conclusion, PUFAs are pivotal in managing intestinal dysbiosis in children due to their anti-inflammatory properties, modulation of gut microbiota, enhancement of gut barrier function, and support of immune health.

### 5.2. Vitamin D

Vitamin D is a lipid-soluble vitamin that is absorbed from dietary sources or supplements in the proximal small intestine [192]. Recent studies have revealed the association between vitamin D deficiency and a multitude of diseases, including IBD [193,194], where vitamin D is intimately involved in the regulation of inflammation via a bidirectional relationship with the gut microbiota [195,196]. Studies also suggest that the amount of dietary vitamin D and its circulating levels may be involved in maintaining immune homeostasis in healthy individuals, partially via modulating the gut microbial composition [197]. Exclusively breastfed infants present a particular risk of vitamin D deficiency, due to its low concentration in breast milk [198], low maternal vitamin D levels, and daily intake, as well as the lack of exposure of newborns and suckling infants to sunlight [199]. Therefore, associations such as the American Academy of Pediatrics and ESPGHAN recommend vitamin D supplementation at doses of 400 IU/day in infants who are exclusively or partially breastfed [195,200]. Regarding vitamin D treatment in IBD pediatric patients, some clinical studies investigated the effectiveness and safety of vitamin D supplementation with the aim of restoring hypovitaminosis D associated with IBD [201,202]. To date, only a few studies investigated the beneficial effects of vitamin D supplementation in children with IBD, reporting a significant decrease in the IBD activity score and biochemical inflammatory markers, such as calprotectin and C reactive protein, and interleukins such as IL-2, IL17, and IL23 [203], but without any consideration regarding the effects on the intestinal microbiota.

### 5.3. Minerals: Zinc

The crucial role of zinc in intestinal function is well documented [204]. Zinc is considered a functional food for maintaining gastrointestinal mucosal function [205] and its deficiency significantly impacts the intestinal mucosa, leading to its degeneration and severe adverse effects. This deficiency causes thinning of the mucus layer and alterations in mucus composition that appear to be caused primarily by disrupted mucin synthesis at the post-translational level, as observed in both animal and human goblet cell studies [206,207]. Studies have shown that children with IBD often have lower levels of zinc compared to healthy peers. This deficiency can be due to malabsorption, increased intestinal loss, and inadequate dietary intake. Although zinc supplementation seems to offer potential benefits for managing these conditions in animal and cell models, investigations of its efficiency in pediatric IBD treatment are still lacking. Consequently, there are no specific guidelines for zinc supplementation in children with conditions like IBD [208]. Currently, there is limited well-documented information on the appropriate dosage for pediatric patients.

## 6. Other Therapies for Pediatric IBD

### 6.1. Fecal Microbiota Transplantation (FMT)

Fecal microbiota transplantation (FMT) has been suggested as a mechanism to restore intestinal bacterial diversity. FMT involves transferring stool from a healthy donor to a recipient with the aim of restoring the balance of gut microbiota and promoting therapeutic effects [209,210,211]. FMT can restore the balance and diversity of beneficial bacteria in the gut microbiota by aligning the recipient’s composition with that of the donor. The efficacy and safety of FMT in pediatric patients have been confirmed in several studies and has been applied for severe conditions such as IBD with presumed underlying intestinal dysbiosis, ulcerative colitis, and Crohn’s disease [212,213,214,215,216]. The difference between probiotic or prebiotic therapies and FMT lies in the breadth of bacterial strains transferred. While probiotics and prebiotics only supplement some bacterial strains, FMT offers a more comprehensive approach by transferring hundreds of strains at once. However, this broad spectrum of bacteria in FMT also poses potential risks. Donors might transfer opportunistic pathogenic bacteria or infections to recipients along with the beneficial strains. This risk underscores the importance of rigorous screening and testing protocols for both donors and recipients in FMT procedures to minimize adverse outcomes.

### 6.2. Vaginal Seeding

During vaginal delivery, newborns are naturally exposed to the mother’s vaginal and intestinal bacteria, which play a crucial role in the early development of the infant’s immune system and gut microbiome [217]. Infants born by CS miss this initial exposure and, in relation to vaginally delivered children, show altered microbiota development [218]. Emerging evidence suggests that cesarean delivery may be a risk factor for the later development of childhood problems such as metabolic and inflammatory diseases [219]. Vaginal microbial transfer, also known as vaginal seeding, is a process where infants born by CS are exposed to the mother’s vaginal microbiota, aiming to mimic the natural microbial exposure that occurs during vaginal birth. The process of vaginal seeding involves swabbing obtained from pregnant women just before delivery, stored and applied to newborns’ lips and then to their entire bodies [220] with a gauze or swab that has been previously incubated in the mother’s vaginal fluids. This is performed shortly after birth to introduce beneficial bacteria to the infant’s skin and mucous membranes, thereby promoting the development of a healthy microbiome. To modify the microbiome of a CS baby, researchers tested wiping these babies with swabs that had been in their mother’s vagina for about an hour before the cesarean birth [221]. They found that this technique could partially restore the microbiota of CS babies. Other researchers investigated the differences in the baby microbiome at birth and again at 6 weeks of age. They found that by 6 weeks, there were no significant differences in the microbiomes based on the mode of delivery [222]. As a result, they concluded that any clinical implications of microbiome differences at birth might be minimal or nonexistent. Concern exists about the potential transmission of pathogenic bacteria or viruses from mother to baby. To date, there is no evidence to support potential harm to the baby. The transmission of some pathogens that may be asymptomatic in the mother could cause serious adverse consequences for infants, including herpes simplex virus, *Chlamydia trachomatis*, *Neisseria gonorrhea*, and group B streptococcus. While the concept is promising, it is still under research, and healthcare professionals have yet to reach a consensus on its safety and efficacy. Vaginal seeding following a CS continues to be an experimental procedure and is not recommended as standard practice. There are currently no established guidelines for maternal screening, existing studies have been limited in scope, and there has been insufficient long-term follow-up to fully assess potential lasting effects.

## 7. Clinical Trials

We organized here, in Table 1, clinical trials with effects on intestinal microbiota cited all over the review, giving particular attention to the population enrolled, treatment, and principal outcomes.

## 8. Conclusions and Future Perspectives

In conclusion, the current body of evidence on the use of dietary supplements in pediatric intestinal dysbiosis remains limited. Although some studies suggest the benefits of probiotics and prebiotics in modulating the gut microbiota, further studies are needed to clarify the efficacy and safety of these interventions in the pediatric population. Additionally, the potential role of emerging supplements such as synbiotics (combinations of prebiotics and probiotics), paraprobiotics (non-viable microbial cells or cell fragments), and postbiotics (microbiota metabolites) warrants future investigations. These novel supplements may offer additional therapeutic options, but their effects and safety profiles need to be thoroughly explored in pediatric populations before they can be recommended for widespread use. Effective management of pediatric intestinal dysbiosis through dietary supplementation requires a comprehensive understanding of the underlying mechanisms of intestinal dysbiosis, the specific needs of the pediatric population, and the interactions of different types of supplements and the gut microbiota. Establishing well-defined clinical guidelines based on robust scientific evidence is crucial for ensuring the safe and effective use of these supplements in children. Overall, while there is potential in using dietary supplements to manage pediatric intestinal dysbiosis, the current evidence base is not yet sufficient to make definitive recommendations. Future research should focus on addressing these gaps to provide clearer guidance for healthcare providers and caregivers in the management of pediatric gut health. 

## Figures and Tables

**Table 1:** Clinical trials.

Reference		Population	Treatment	Effects
Ben, X. et al. [69]	Prebiotics	371 Term infants	Infants 4 weeks after birth randomly assigned to IF or IF + GOS for 3 months	HM and IF + GOS intestinal *Bifidobacteria*, *Lactobacilli* 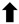 Fecal pH 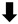
Prieto, P.A. et al. [70]	Prebiotics	84 Term infants	Infants within 11 days after birth, randomly assigned to IF or IF + FOS for 16 weeks	HM and IF + FOS Intestinal *Lactobacilli* 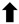
Puccio, G. et al. [71]	Prebiotics	175 Term infants	Infants within 14 days after birth, randomly assigned to IF or IF + 20FL + LNnT for 6 months	IF + 20FL + LNnT safe and well tolerated, morbidity (bronchitis) and medication 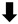 use (antipyretics and antibiotics)
Miele, E. et al. [92]	Probiotics	29 patients (mean age: 9.8 years; female/male: 13/16),	Children newly diagnosed for UC, randomly assigned to VSL#3 weight-based dose, (range: 450–1800 billion bacteria/day) (n = 14) or placebo (n = 15) *	Endoscopic, histological scores 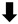 No biochemical or clinical adverse events
Huynh, H.Q. et al. [93]	Probiotics	18 patients (mean age: 12.2 years; female/male: 7/11),	All UC patients received 3 g sachet of VSL#3 twice daily by mouth for 8 Weeks	10 patients remission (SCCAI < 3); 1 patient response (decrease in SCCAI 2, final score 5); 7 patients no changes. Bacterial taxonomy changes VSL#3 well tolerated No adverse effects
Pietrzak, A. et al. [161]	Postbiotics	72 patients (mean age: 13.5 years; female/male: 14/28): 42 Crohn’s disease, 30 mild conditions	Randomly assigned to sodium butyrate 150 mg twice a day for 12 weeks (n = 29) or placebo (n = 23)	Not effective as an adjunctive treatment
Kunde, S. et al. [213]	FMT	9 patients (7–21 years) Mild to moderate UC	Freshly prepared fecal enemas daily for 5 days	7 patients response within 1 week, 6 patients maintained response at 1 month. No adverse effects, good tolerability
Wilson, B.C. et al. [220]	Vaginal seeding	47 newborns 22 Vaginal delivery (control) 12 Cesarean-seeded 13 Cesarean placebo	Newborns randomized to 3 mL solution of maternal vaginal microbes or sterile water; stool samples at 1 h, 1 month, and 3 months undergoing shotgun metagenomic sequencing	No differences in gut microbiome composition or functional potential were observed

HM (Human milk), IF (Infant Formula), FOS (fructooligosaccharide), GOS (galacto-oligosaccharides), 20FL (1.0 g/L 20fucosyllactose), LNnT (0.5 g/L lacto-N-neotetraose), * Concomitant steroid induction and mesalamine maintenance treatment for both groups, 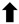 = increase, 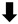 = decrease.

## Data Availability

Data sharing not applicable.
